# A model of abusive supervision, self-efficacy, and work engagement among Chinese registered nurses: The mediating role of self-efficacy

**DOI:** 10.3389/fpsyg.2022.962403

**Published:** 2022-11-29

**Authors:** Ning Sun, Qiulan Zheng, Laiyou Li, Haibo Zhu, Xiufen Liu, Shuping Zhou, Huihui Han

**Affiliations:** ^1^Ningbo College of Health Sciences, Ningbo, China; ^2^The second affiliated hospital of Chongqing Medical University, Chongqing, China; ^3^The second affiliated hospital of Heilongjiang University of Chinese Medicine, Harbin, China; ^4^The affiliated people’s hospital of Ningbo University, Ningbo, China

**Keywords:** abusive supervision, self-efficacy, work engagement, nursing managers, leadership

## Abstract

Abusive supervision could negatively influence individual work attitudes, behaviors, and work outcomes. Self-efficacy and work engagement can help to increase nursing performance. But few studies have attempted to determine the specific mechanism between them in China. The objective is to analyze the levels of abusive supervision, self-efficacy, and work engagement, and to explore the relationship between these three variables among Chinese clinical registered nurses. A predictive, cross-sectional quantitative survey was performed in a convenience sample of 923 Chinese clinical nurses. The instruments included the Demographic Data Questionnaire, Abusive Supervision Scale, Self-efficacy Scale and Work Engagement Scale. A total of 702 valid questionnaires were returned, yielding a favorable response rate of 76.1%. The level of abusive supervision was at the mid-low level, with a mean of 1.55. The nurses presented a relative high level of self-efficacy (M = 4.97) and work engagement (M = 5.01). A statistically significant negative correlation between abusive supervision and self-efficacy (*r* = −0.21, *p* < 0.01). A statistically significant negative correlation between abusive supervision and work engagement (*r* = −0.32, *p* < 0.01), and a statistically significant positive correlation between self-efficacy and work engagement (*r* = 0.43, *p* < 0.01). Abusive supervision had a directly negative effect on self-efficacy (*β* = −0.23, *p* < 0.01) and work engagement (*β* = −0.24, *p* < 0.01). Self-efficacy positively predicted work engagement (*β* = 0.41, *p* < 0.01). The results indicated that abusive supervision could negatively predict nurses’ work engagement directly and that abusive supervision could also indirectly influence work engagement partly through the mediation of self-efficacy. Nursing managers should take effective measures to prevent and control the abusive management and leadership behavior of head nurses, and improve nurses’ self-efficacy, so that nurses can experience full respect, support, and self-confidence. They can devote themselves to work with the greatest enthusiasm.

## Introduction

The current global nursing shortage has been concerning, particularly for nursing managers. According to the latest data from the National Health and Medical Commission of China, there were 5.018 million nurses in China. There were 3.56 registered nurses per 1,000 population and a medical-to-nursing ratio of 1:1.17 at the end of 2021. There is a million-level gap in the number of nurses in China. Especially in recent years, the continuous outbreak of the new crown pneumonia epidemic has highlighted the problems of nurses’ job burnout, willingness to leave, and psychological exhaustion etc. Hence, it is imperative to maximize limited nursing human resources to ensure that nursing quality remains high in China (Central People's Government of the People's Republic of China, 2022). Based on the literature review, the study is based on Bakker’s job requirement-resource model (JDR). The model assumes that the level of work engagement depends on the balance of work requirements and work resources. Job demands require the individual to make constant physical, mental, cognitive, and emotional efforts to complete various aspects of the work, which may cause adverse physical or psychological stress. Work resources can offset the adverse effects of job requirements, effectively stimulate employees’ work motivation, improve their work engagement level, and produce positive work results (Bakker and Demerouti2007). With the development of positive psychology, nursing researchers have focused on developing psychological nursing resources, such as psychological empowerment, psychological capital, self-efficacy, work engagement, etc.(Lin et al., 2020) in China. Work engagement has been proven to improve job satisfaction (Waltz et al., 2020), retention (Gustafsson et al., 2021), and nurses’ work ability (Tomietto et al., 2019), which significantly influences nursing outcomes. Meanwhile, evidence has shown that authentic leadership (Lisbona et al., 2021) and transformational leadership (Wu and Lee, 2020; Schaufeli, 2021) are important factors that influence nurse work engagement. However, destructive behaviors are ignored by most nursing managers, such as abusive supervision. Abusive supervision could negatively influence individual work attitudes, behaviors and work outcomes (Qian et al., 2015; Özkan, 2021). However, few studies have examined the relationship between abusive supervision and work engagement among nurses in China. Furthermore, the literature also indicates that the intrinsic motivational process of work engagement is explained by self-efficacy (Wallin et al., 2021), but few studies have attempted to determine the specific mechanism underlying this process in China. Therefore, this study aimed to explore the relationships between abusive supervision, self-efficacy, and work engagement; we also sought to determine the mechanisms that might explain the links between these three variables in China.

## Background

Abusive supervision refers to subordinates’ perceptions of the extent to which supervisors engage in the sustained display of hostile verbal and nonverbal behaviors, excluding physical contact (Finlay et al., 2020). Behavioral descriptions of abusive supervision include the silent treatment, impoliteness, aggressive eye contact, angry tantrums, explosive outbursts (e.g., yelling at someone for disagreeing), intimidation (e.g., threats of job loss), and derogatory words, as well as ridiculing or humiliating someone in front of others (DeMarce et al., 2021). Some studies have linked abusive supervision to organization attitudes, behaviors, and work outcomes, indicating that abusive supervision has some deleterious consequences as follows. Baig and Riaz (2021) have reported that abusive supervision is positively associated with stress and emotional exhaustion. Other scholars have also found that abused subordinates may experience anxiety (Huang et al., 2019), and insomnia (Sannes et al., 2021). A statistically significant negative correlation between abusive supervision and organizational citizenship behavior (OCB) has been observed by Baig and Riaz (2021). When subordinates have the perception of being abused, they might refuse to help coworkers, exhibit negative attitudes that affect their ability to finish their work, and even reduce their chances of receiving earned rewards (Estes, 2013). Some researchers have also sought to explore abuse in the nurse supervisor-subordinate relationship. In Estes (2013) study, 46.6% of the nurses investigated reported experiencing abusive supervision, and 36.6% even thought that their performance was negatively influenced by abusive supervision. Sharif Nia et al. (2021) have suggested that the increasing occurrence of abusive supervision can be harmful for individuals and organizations, including decreased job satisfaction and increased adverse events.

Work engagement is defined as “a positive, fulfilling, work-related state of mind that is characterized by vigor, dedication, and absorption in the activity”(Waltz et al., 2020). In recent years, most studies have confirmed that work engagement can significantly predict positive outcomes. Particularly in healthcare settings, work engagement is becoming an important resource to enhance nursing recruitment and retention (Gustafsson et al., 2021), increase commitment (Lin et al., 2020), and advance performance (Eguchi et al., 2020). Therefore, exploring determinants of nurses’ work engagement has great significance. In terms of the links between leadership level and nursing work engagement, Tillott et al. (2013) have claimed that management factors were relatively important to others in influencing work engagement. García-Sierra and Fernández-Castro (2018) empirically demonstrated that transformational leadership is positively related to work engagement but that organizational justice mediated their relationship, meaning that managers’ leadership significantly predicts nurses’ perception of justice and, eventually, influences work engagement. In addition, a statistically significant positive correlation between abusive supervision and work burnout was found by Carlson et al. (2012). Scholars have hypothesized that work engagement is the opposite of burnout (Koranne et al., 2022). Work burnout and work engagement are opposites along two distinct bipolar dimensions. Hence, nurses who are exhausted are less likely to engage in their work.

Self-efficacy refers to “people’s judgments of their capabilities to organize and execute the courses of action required to attain designated types of performances” (Bandura, 1986). As a type of important personal resource, self-efficacy has been demonstrated as having vital and precursor influences on positive consequences. Tsai et al. (2014) have suggested that nurses with high self-efficacy had higher professional commitment and invested more effort into their jobs. Enhanced self-efficacy can help to improve the quality of nursing care and increase nursing performance (Eller et al., 2018; Kim et al., 2021). Wallin et al. (2021) have also claimed that self-efficacy, as the main personal resource, explains intrinsic motivational processes, such as work engagement. Accordingly, we consider that the higher a nurse’s self-efficacy, the more likely the nurses engage in the nursing professional practice autonomously. According to social cognitive theory, vicarious experiences and verbal persuasion, as a contextual resource, strongly influence individual self-efficacy (Bandura, 1986). Salanova et al. (2011) have claimed that managers can effectively increase self-efficacy among nurses through role modeling and verbal encouragement. However, if the managers tend to demonstrate abusive supervision, open verbal communication and bi-directional feedback will be destroyed. Hence, we believe that abusive supervision can negatively predict self-efficacy. In addition, as stated earlier, abusive supervision can cause stress, emotional exhaustion, burnout, and depression, and then decrease the motivation to engage in the job (Baig and Riaz, 2021). In the process, self-efficacy can efficiently regulate the predicament to achieve a desired balance between work attitude and behavior. For these reasons, we believe that abusive supervision will influence nurses’ work engagement by mediating self-efficacy.

### Aim of the study

The aim is to analyze the levels of abusive supervision, self-efficacy, and work engagement and to explore the relationship between these three variables among Chinese clinical registered nurses. Four hypothesizes were posed as follows:

1. There is a negative relationship between abusive supervision and work engagement.
2. There is a positive relationship between self-efficacy and work engagement.
3. There is a negative relationship between abusive supervision and self-efficacy.
4. Abusive supervision can indirectly predict work engagement by mediating self-efficacy.

### Methods

#### Study design

A correlational, cross-sectional design was adopted, and questionnaires were used for data collection.

#### Setting and sample

The study was performed in four hospitals in Harbin, China over an 8-month period in 2020 and 2021. A convenience sampling was used to survey full-time registered nurses. A total of 923 nurses agreed to participate in the study, and 702 completed questionnaires were usable, with a response rate of 76.1%. Participation was voluntary and anonymous. All study participants were full-time registered nurses who had been employed as clinical care staff nurses for at least 1 year. Samples with primary disease were excluded.

#### Sample size calculation

The study mainly examined the correlation among abusive supervision, self-efficacy, and work engagement. Multi-factor analysis methods were applied. Based on relevant research, it was estimated that 49 variables could be entered into the model. The required sample size was estimated to be at least 10–15 times the number of variables entered into the model, thus requiring 735 research subjects. The follow-up loss rate was calculated at 10%; thus, the sample size needed was about 810 people.

#### Data collection

Two investigators underwent unified training. Data were collected by face-to-face interviews. All participants were included in this study after a scheduled meeting arranged by the nursing managers in the hospital. The investigators explained the research objectives and methods to individuals who met the inclusion criteria and obtained consent and cooperation from participants. Consenting participants received an envelope containing a packet with the questionnaires. Participants completed the questionnaires immediately upon receipt and placed them in the envelope for collection by the investigators. To ensure anonymity, each completed questionnaire was assigned a code number.

#### Instruments

Four structured questionnaires were used for the data collection.

##### The demographic data questionnaire

The Demographic Data Questionnaire was designed by researchers, and it contains information about gender, age, marital status, department, education background, job title, and tenure.

##### The abusive supervision scale

Abusive supervision was measured using abusive supervision scale developed by Tepper (2000). The Chinese version was used after formal discussion and reliability testing by Chinese researchers, with a translation consistency of 0.92, test–retest reliability of 0.76, content validity of 0.95, and internal consistency of 0.95(Li et al. 2014). The scale contains 15 items (e.g., “my direct supervisor discloses my past mistakes and failures”) and uses a 5-point Likert scale ranging from 1 (never) to 5 (very often). A higher score indicates more abusive supervision that the nurses perceived. In the study, the overall reliability for the scale was 0.96.

##### The self-efficacy scale

Self-efficacy was measured by Jia and Li (2010) who performed a translation and back-translation process and created the final Chinese version after formal discussion and reliability testing, with a translation consistency of 0.93, test–retest reliability of 0.71, content validity of 0.99, and internal consistency of 0.81. The scale consists of 17 items and uses a 6-point Likert scale ranging from 1 (strongly disagree) to 6 (strongly agree). Six items (e.g., “Failure will only make me work harder”) are positively scored, and the others (e.g., “It’s easy for me to give up”) are reverse scored. A high score indicates a higher level of self-efficacy.

##### The Utrecht Work Engagement Scale

Work engagement was measured using the Utrecht Work Engagement Scale (Schaufeli et al., 2002). The Chinese version was used after formal discussion and reliability testing by Chinese researchers, with a translation consistency of 0.90, test–retest reliability of 0.78, content validity of 0.96, and internal consistency of 0.93 (Zhang and Gan, 2005). The scale contains three dimensions (17 items), namely vigor (6 items, e.g., “At work, I feel bursting with energy”), absorption (6 items, e.g., “I feel time passed quickly, when I’m working”), and dedication (5 items, e.g., “I am enthusiastic about my job”). Answers were given on a 7-point Likert scale ranging from 1 (strongly disagree) to 7 (strongly agree). In the study, the overall reliability for the scale was 0.94.

### Ethical considerations

This study and the gathering of data were approved by the Harbin Medical University Institutional Review Board. All participants provided written informed consent prior to participating in this study. Two researchers were responsible for informing participants, both in writing and orally, about the purpose of the study and the data gathering. The participants were informed that the survey was completely voluntary and that withdrawal from the study was available at any time, without any negative repercussions. Each nurse who consented to take part was asked to complete the survey within a week after receiving an envelope containing a packet with the four questionnaires. To minimize misgivings and pressure perceived by nurses, we used anonymous questionnaires, and we asked each participant to place their completed questionnaires into the envelope and seal it. Moreover, the completed questionnaires returned were uniformly coded by researchers to ensure anonymity. All nurses were anonymized and guaranteed confidentiality.

### Data analysis

SPSS Statistics 22.0 and AMOS Graphics 21.0 (IBM Corporation, New York, United States) were used for the statistical analyses. First, SPSS 22.0 was used to conduct descriptive statistics and Pearson’s correlations. Then, AMOS 21.0 was used to conduct structural equation modelling (SEM). We used the maximum likelihood estimation method in SEM to confirm our hypothesis and to link the relationships between abusive supervision, self-efficacy, and work engagement. The goodness-of-fit of model was evaluated through relative and absolute indices (Marsh et al., 1996). In the study, the absolute indices that were calculated include normed the Chi-squared (χ^2^/df) test, goodness-of-fit Index (GFI), adjusted goodness-of-fit index (AGFI), root mean square error of approximation (RMSEA), and root mean square residual (RMR). The following relative indices were used: the comparative fit index (CFI), normed fit index (NFI), incremental fit index (IFI) and Tucker Lewis index (TLI). Bollen (1989) believed that a value lower than 3.0 for a normed Chi-squared test indicates a good model fit. GFI, CFI, NFI, IFI, and TLI values greater than 0.90 indicate an acceptable model fit. Finally, the RMSEA and RMR should be less than 0.08 (Kline, 2005).

## Results

### Participant demographics

A total of 93.2% (654) of the nurses are female, and the rest (6.8%, 48) are male. The participants’ average age was 28.00 years (SD = 5.18 years; range, 20 to 52 years). The average job tenure was 6.15 years (SD = 5.68 years; range, 1 to 34 years). Married The nurses were mainly single (54.4%,389). A total of 43.4%(305) of the nurses work in medicine. The nurses were mainly bachelor education (69.1%,485). The job title was mainly nurses (49.9%0.350). The detailed demographics are presented in Table 1.

**Table 1:** Respondent demographics (*n* = 702).

Variable	Type	Nurses	Percentage (%)
Years of age	20–25	252	35.9
	26–30	306	43.6
	30–35	90	12.8
	≥36	54	7.7
Years of experience in nursing	1–2	187	26.6
	3–5	231	32.9
	6–8	131	18.7
	≥9	153	21.8
Marital status	Married	306	43.6
	Single	389	54.4
	Divorced	7	1
Department	Medicine	305	43.4
	Surgery	222	31.7
	Operating Room	28	4
	ICU	49	7
	Other	98	14
Education background	Technical Secondary School	11	1.6
	Junior College	193	27.5
	Bachelor	485	69.1
	Master or Above	13	1.9
Job title	Nurse	350	49.9
	Junior Nurse	260	37.0
	Senior Nurse	87	12.4
	Associate Superintendent Nurse	5	0.7

### Descriptions and correlations

The means, standard deviations, Cronbach’s alpha coefficients, and correlations for the study variables are presented in Table 2. In the present study, all scales used showed adequate levels of reliability from 0.81 to 0.96 (see Table 2). Overall, the level of abusive supervision that nurses perceived was at the mid-low level, with a mean of 1.55. The nurses presented a relative high level of self-efficacy (M = 4.97, above average > 3.5). The score of work engagement (M = 5.01) was slightly higher than the median of 4. Meanwhile, nurses thought that they had the highest level of dedication (M = 5.35) and the lowest level of absorption (M = 4.96) at work. In addition, we found a statistically significant negative correlation between abusive supervision and self-efficacy (r = −0.21, *p* < 0.01), a statistically significant negative correlation between abusive supervision and work engagement (*r* = −0.32, *p* < 0.01), and a statistically significant positive correlation between self-efficacy and work engagement (*r* = 0.43, *p* < 0.01; see Table 2).

**Table 2:** The means, standard deviations, alpha reliabilities and correlations for the study variables.

Variable	Range	Mean	SD	Alpha	1	2	3	4	5	6
1.Abusive supervision	1–5	1.55	0.71	0.96	1					
2.Self-efficacy	1–6	4.97	0.70	0.81	−0.21^**^	1				
3.Work engagement	1–7	5.01	1.26	0.94	−0.32^**^	0.43^**^	1			
4.dedication	1–7	5.35	1.30	0.82	−0.29^**^	0.46^**^	0.93^**^	1		
5.absorption	1–7	4.96	1.38	0.87	−0.31^**^	0.37^**^	0.96^**^	0.84^**^	1	
6.vigor	1–7	4.74	1.31	0.82	−0.30^**^	0.40^**^	0.94^**^	0.80^**^	0.85^**^	1

** Statistically significant correlations at 0.01 (two-tailed test).

### Hypotheses testing

We tested the hypotheses through structural equation modeling. In the model, abusive supervision served as an exogenous latent variable, and self-efficacy and work engagement served as endogenous latent variables. Specifically, work engagement had three observed variables (i.e., dedication, absorption, and vigor). Considering that abusive supervision and self-efficacy were single dimension constructs, we used the item parceling approach to divide their items into three (i.e., A-P1, A-P2, and A-P3) and four (i.e., S-N1, S-N2, S-N3, and S-N4) observed variables, respectively, and then reduced the indicator numbers and improved the modeling efficiency. In the model building, we included paths from abusive supervision to self-efficacy and work engagement, and from self-efficacy (as the mediator) to work engagement. The χ^2^/df was lower than 3. The GFI, AGFI,CFI, RFI, NFI, IFI, and TLI were all higher than 0.90. The RMSEA and RMR were all lower than 0.05. These values indicate a good model fit. According to the fit indices, our hypothesized model was fitted to the data (see Table 3).

**Table 3:** Model fit indices.

Index	χ^2^	d.f.	χ^2^/d.f.	GFI	AGFI	RMSEA	CFI	RFI	NFI	IFI	TLI	RMR
Research Model	81.305	32	2.541	0.978	0.962	0.047	0.991	0.991	0.985	0.991	0.987	0.027
criteria			<3	>0.9	>0.9	<0.05	>0.9	>0.9	>0.9	>0.9	>0.9	<0.05

d.f., degrees of freedom; χ^2^/d.f., normed Chi-squared; GFI, goodness-of-fit index; AGFI, adjusted goodness-of-fit index; RMSEA, the root mean square error of approximation; CFI, comparative fit index; NFI, normed fit index; RFI, relative fit index; IFI, incremental fit index; TLI, Tucker–Lewis Index; RMR, root mean square residua.

**Figure 1:**
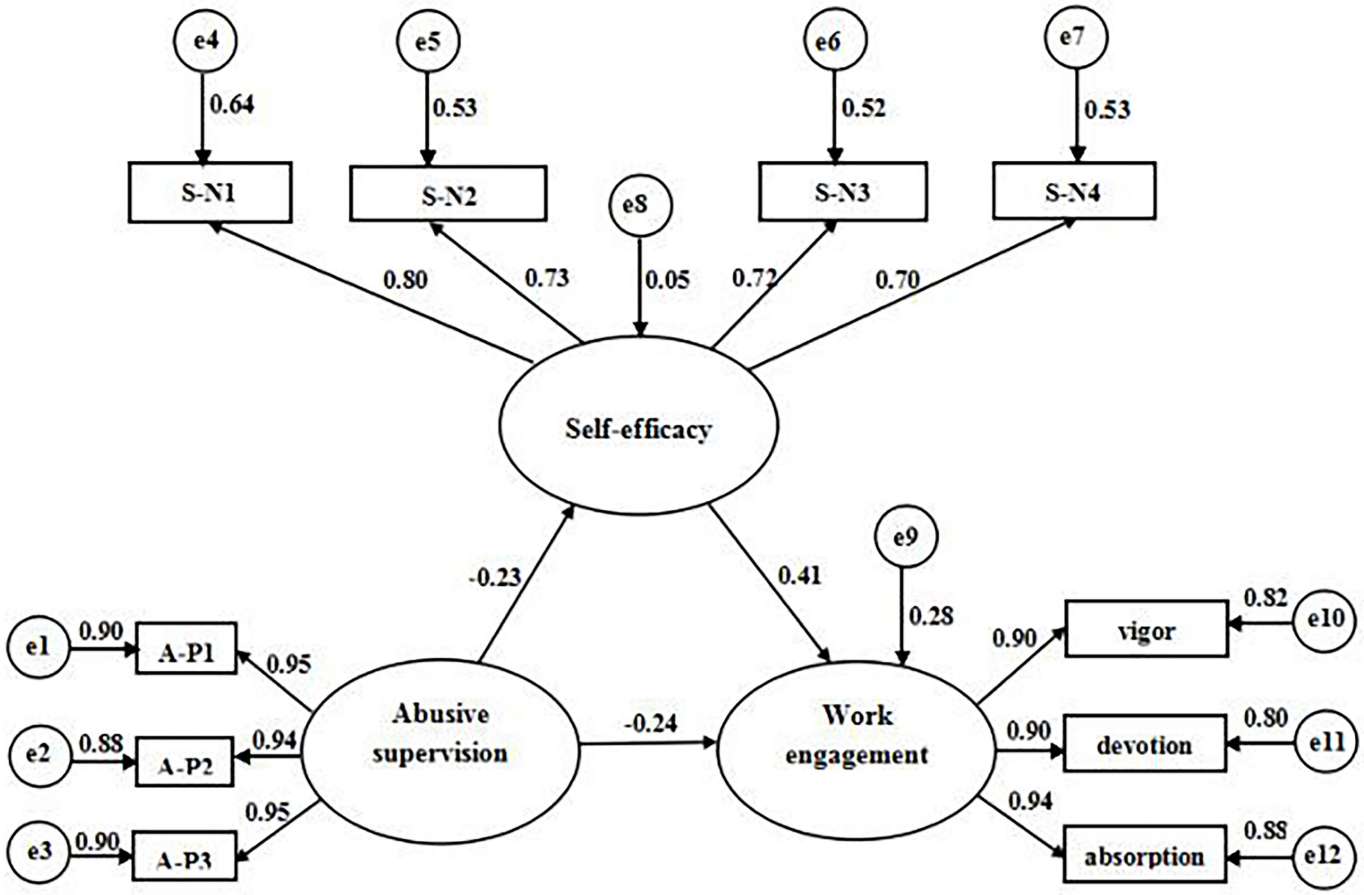
The study model with standardized path coefficients. Note 1. All coefficients were statistically significant (*p* < 0.01). Note 2. A-P1, A-P2 and A-P3 = parceled items that make up the abusive supervision scale. S-N1, S-N2, S-N3, and S-N4 = parceled items that make up the self-efficacy scale.

All parameter estimates and path coefficients were statistically significant (see Figure 1). The final model demonstrated that abusive supervision had a directly negative effect on self-efficacy (*β* = −0.23, *p* < 0.01) and work engagement (*β* = −0.24, *p* < 0.01). Self-efficacy also positively predicted work engagement (*β* = 0.41, *p* < 0.01). Overall, abusive supervision explained 5% of the variance in self-efficacy and 6% of work engagement. Self-efficacy explained 17% of the variance in work engagement. The bootstrapping approach was also used to further test the model. The indirect effect of the mediation model was shown in Table 4. The significance of indirect effect demonstrated the validity of the mediation model.

**Table 4:** The indirect effect, direct effect, and total effect of the mediation model.

Path	Effect size	S.E.	*p*-Value	Bootstrap 95%CI		Effect proportion (%)	*R* ^2^	Lower	Upper
Indirect effect	−0.16	0.04	<0.001	−0.25	−0.09	28.07	0.28
Direct effect	−0.41	0.07	<0.001	−0.56	−0.27	71.93	
Total effect	−0.57	0.06	<0.001	−0.70	−0.45		

## Discussion

The results showed that the average score of abusive management was 1.55 ± 0.71. The item with the highest score was that my leader would criticize subordinates in front of everyone, indicating that nurses pay more attention to the head nurse’s verbal evaluation and feedback on themselves. When the head nurses educate their subordinates, they usually ignore the inner feelings of the nurses. The results showed that the self-efficacy score of nurses was 4.97 ± 0.70. The item with the highest score was that when encountering difficulties at work, nurses were willing to try to solve the problem, and showed good confidence in their ability to deal with work problems. The results show that nurses have strong self-belief and are more confident in their professional skills when faced with complex and numerous nursing work. The average score of nurses’ work engagement was 5.01 ± 1.26, which was at an upper-middle level, similar to the findings of Wang et al. (2012). It shows that nurses are more active in performing nursing work. Among the three dimensions of work engagement, the average score of dedication is the highest, and the average score of vitality is the lowest (Table 2). The level of work engagement of Chinese nurses is lower than that of Europe and the United States (Jin et al. 2022), which suggests that effective measures should be taken, especially in terms of improving work vitality. Improving the work engagement level of nurses is an urgent problem that needs to be addressed by managers in China.

Our study aimed to explore the relationships between abusive supervision, self-efficacy and work engagement, and to extend the knowledge of the mechanisms through which abusive supervision predicts nurses’ work engagement. Our results were similar to the findings of Sharif Nia et al. (2021), likely because most Chinese nursing managers (i.e., charge nurses) are females. Influenced by traditional culture, Chinese females are characterized by subtle emotion and sensibility. When resolving their relationships with sub-nurses, they are more likely to be convergent in abuse and, in turn, to emotionally encourage nurses. However, given that abusive supervision is steadily increasing and has detrimental effects on individuals and organizations (Sharif Nia et al., 2021), attention directed toward this issue should be continuously increased. In addition, self-efficacy and work engagement scores are consistent with previous nursing studies conducted by Eller et al. (2018) and Kim et al. (2021), respectively, indicating considerable similarities.

The findings demonstrate several theoretical and practical implications. First, as shown in hypothesis 1, the perceived abusive supervision by nurses negatively predicts work engagement. This finding is supported by the JDR (Estes, 2013) (i.e., abusive supervision replaces job recourse, such as supervisor support and positive feedback, and, in turn, increases nurses’ burden in job demand, which eventually has a detrimental effect on work engagement). For example, when nurses perceived they are suffering from abuse, for instance, being told that they have stupid ideas and are incompetent, they can be disturbed by the lack of manager support and encouragement. Then, the nurses’ work enthusiasm is greatly reduced. Our findings also align with those of Carlson et al.(Carlson et al., 2012), who demonstrated that abusive supervision contributes to work burnout and, in turn, decreases work engagement level.

Second, our research confirmed the motivational process of work engagement based on Bandura’s social cognitive theory (i.e., hypothesis 2 (Bandura, 1986). The theory indicates that the individual’s attitudes and behaviors are predicted by efficacy expectations. Higher levels of self-efficacy and confidence align with a stronger intrinsic motivation. The results are consistent with those of Eller et al. (2018), who identified that high self-efficacy can help nurses enhance considerable work engagement, thereby improving extra-role performance in Western countries. Therefore, we deeply believe that to achieve nursing job goals, nurses with high level self-efficacy are more likely to undertake the task actively, make unremitting endeavors, and devote themselves to their jobs.

Third, as hypothesis 3 predicted, we argued that the more abusive the behaviors conducted by nursing supervisors, the lower self-efficacy is experienced by nurses. According to Bandura (1986) suggestion, the process of individual’s behavior motivation (i.e., self-efficacy) was dependent on both accepting others’ verbal persuasion and observing behavioral models. In China, the charge nurse, as the first-line manager in the ward, serves as the backbone, guiding the nursing activities and serving as a role modeling for nurses. When nurse managers continuously behave in an abusive manner, such as criticizing nurses or denying nurse’s abilities, the nurses are likely to experience depression and self-doubt, and then they lack of confidence in their jobs and eventually present with decreased self-efficacy. Worse still, this abusive leadership style, as a salient example, can cause individuals to mirror that behavior and treat other team members with aggression and hostility (Huang et al., 2019).

Finally, the most compelling finding of our research is the mediating role that self-efficacy plays between abusive supervision and work engagement (hypothesis 4).

This result lends support to the notion that when nursing managers present abusive behaviors, nurses will experience less self-efficacy in their jobs, which then decreases their work engagement. The results are similar with Wallin et al. (2021) which pointed out that nurses’ self-efficacy plays a mediating role between leadership behavior and work engagement. Zhang and Li (2015) pointed out that nurses’ work effort depends to a certain extent on leader factors, and work motivation (such as self-efficacy) is a powerful contributing factor for them to achieve work goals. At the same time, the Chinese researcher also emphasized that nurses with higher self-efficacy had more self-confidence, they were more proactive in dealing with stressful events, focused on their work with more enthusiasm, and their level of work engagement will be higher (Li, 2021). These studies explain the mediating process of self-efficacy as a motivational variable. Therefore, the study indicated that abusive management as a harmful leadership behavior will make nurses continue to be confused and questioned about their own abilities, thereby blocking their intrinsic motivation process, resulting in powerlessness and frustration, and ultimately affecting their work vigor, focus, and dedication. This suggests that in the process of management, head nurses should not only pay attention to restraining their abusive leadership behaviors, but also learn to use relevant theories to stimulate nurses’ self-efficacy, so as to better stimulate the inner potential of nurses and obtain a high level of work engagement team in China.

### Implication

#### The practical implication

As presented in the introduction, abusive supervision has many negative effects on individuals and organizations. Particularly in the healthcare setting, nurses face different types of stress, and they have to take certain risks in caring for patients. If the nurses are abused frequently, they would expend more energies to address these stresses and their self-efficacy in nursing professional development may be adversely influenced. Different from Western countries, Chinese nurses’ stresses primarily stem from their high workload (usually one nurse to dozens of patients), the tense relationship between nurses and patients (medical injuries have frequently occurred in recent years), continuous night shifts, low income levels, and other social factors. Additionally, when nurses are often long exposed to abuse, their work enthusiasm and values that were present when they chose the occupation are easily exhausted. In turn, as predicted in our study, the lower self-efficacy contributes to inconsiderable vigor, dedication, and absorption. Therefore, nursing managers should take the consequences of abusive supervision seriously, and they ought to take effective measures to prevent and control abusive leadership.

#### The theoretical implication

A comprehensive strategy should be adopted to intervene the abusive management behavior of head nurses. It is a great significance in promoting nurses’ self-efficacy and work engagement level. First, in the process of selecting the head nurse, the selectors need to fully and comprehensively evaluate the candidate’s personality characteristics, management philosophy, and leadership style excluding those who are abusive. Second, most head nurses in China are dominated by experiential management, lacking professional and systematic management learning and training. The head nurses require to learn relevant management theories and application experience. Therefore, the hospital needs to provide management training and leadership development opportunities for head nurses.

### Limitations and future research

The first limitation of the present study is that we used convenience samples and self-reports; thus, common method variance could bias our results. Therefore, we suggested a longitudinal multi-source study to test the causal effects of the variables. Second, the data we obtained were only from Northeast China; therefore, the results may not be generalized to the entire country. Thus, it would be interesting for researchers to involve a larger sampling range to check the proposed model in our study. Third, the abusive supervision scale was developed in a non-nursing profession, and it was not professionally sensitive in a nursing context. Therefore, future research should explore a more specific instrument for measuring abusive supervision in nursing environment. Moreover, to draw a stronger conclusion, an interviewing method can be helpful to understand the underlying mechanisms of the links between abusive supervision and its consequences. Fourth, we only examined the relationship between abusive supervision and work engagement through self-efficacy. Other outcomes of abusive supervision should be considered in the future. Finally, an intervention study design would be interesting in future studies.

## Conclusion

The findings of our study confirm a statistically significant structural association between abusive supervision, self-efficacy, and work engagement among Chinese nurses. The findings further indicate that abusive supervision negatively predicted work engagement, and self-efficacy emerged as an important mediating factor. This finding implies that preventing and controlling abusive leadership behaviors, while comprehensively improving nurses’ self-efficacy level, might have vital significance in contributing to work engagement. If hospital managers take positive actions to select, train, and monitor nursing supervisors and strive to build a healthy, polite, and respectful work environment that pays attention to encourage nurses’ confidence in their abilities, they can achieve more work engagement and increase the loyalty of nurses to their organizations.

## Data availability statement

The raw data supporting the conclusions of this article will be made available by the authors, without undue reservation.

## Ethics statement

This study and the gathering of data were approved by the Harbin Medical University Institutional Review Board. All participants provided written informed consent prior to participating in this study.

## Author contributions

NS and LyL: conception, design, analysis, and data interpretation, drafting the manuscript, revising the manuscript, and its final approval. QlZ and XfL: acquisition of data, project administration, manuscript revisions, and its final approval. XfL and SpZ: formal analysis, manuscript revision, and final approval. LyL and HhH: conception, manuscript revision, and final approval. NS: conception, design, funding acquisition, project administration, manuscript revision, and final approval. All authors contributed to the article and approved the submitted version.

## Conflict of interest

The authors declare that the research was conducted in the absence of any commercial or financial relationships that could be construed as a potential conflict of interest.

## Publisher’s note

All claims expressed in this article are solely those of the authors and do not necessarily represent those of their affiliated organizations, or those of the publisher, the editors and the reviewers. Any product that may be evaluated in this article, or claim that may be made by its manufacturer, is not guaranteed or endorsed by the publisher.

## Abbreviations

SEM: Structural equation modelling
GFI: Goodness-of-fit Index
AGFI: Adjusted goodness-of-fit index
RMSEA: Root mean square error of approximation
RMR: Root mean square residual
CFI: Comparative fit index
RFI: Relative fit index
NFI: Normed fit index
IFI: Incremental fit index
TLI: Tucker–Lewis index
